# MedGAN: optimized generative adversarial network with graph convolutional networks for novel molecule design

**DOI:** 10.1038/s41598-023-50834-6

**Published:** 2024-01-12

**Authors:** Bruno Macedo, Inês Ribeiro Vaz, Tiago Taveira Gomes

**Affiliations:** 1 Faculty of Medicine, University of Porto, Porto, Portugal; 2 Department of Community Medicine, Information and Decision in Health, Faculty of Medicine, University of Porto, Porto, Portugal; 3 Center for Health Technology and Services Research (CINTESIS), Porto, Portugal; 4 Faculty of Health Sciences, University Fernando Pessoa, Porto, Portugal; 5 SIGIL Scientific Enterprises, Dubai, UAE; 6 MedFacts Lda., Lisbon, Portugal

**Keywords:** Drug discovery, Molecular biology, Molecular medicine, Computational biology and bioinformatics, Machine learning

## Abstract

Generative Artificial Intelligence can be an important asset in the drug discovery process to meet the demand for novel medicines. This work outlines the optimization and fine-tuning steps of MedGAN, a deep learning model based on Wasserstein Generative Adversarial Networks and Graph Convolutional Networks, developed to generate new quinoline-scaffold molecules from complex molecular graphs, including hyperparameter adjustments and evaluations of drug-likeness attributes such as pharmacokinetics, toxicity, and synthetic accessibility. The best model was capable of generating 25% valid molecules, 62% fully connected, from which 92% were quinolines, 93% were novel, and 95% unique, preserving chirality, atom charge, and favorable drug-like properties while generating 4831 novel quinolines. These results provide valuable insights into how activation functions, optimizers, learning rates, neuron units, molecule size and constitution, and scaffold structure affect the performance of generative models and their potential to create new molecular structures, enhancing deep learning applications in computational drug design.

## Introduction

The urgency for new and effective drugs is becoming increasingly pressing in modern medicine across various classes of drugs, such as antibiotics, owing to the emergence of drug-resistant bacteria, cancer treatments, tumor heterogeneity, and neurodegenerative conditions, hindered by complexities in drug delivery and understanding disease mechanisms, autoimmune disorders, reflecting complex immune system targeting, and antiviral treatments, complicated by rapid mutation rates and latent viral reservoirs^1–4^. This evolving landscape emphasizes the urgent requirement for new therapeutic agents, calling for a renewed focus and investment in research and development to stay ahead of this critical healthcare need.

Drug discovery is a complex and time-consuming process that requires exploration of a vast chemical space. In living systems, proteins and small molecules constitute a very small portion of all possible small carbon-based compounds. The exploration of this chemical space could lead to the discovery of novel and potentially transformative drugs^5^.

Estimates suggest that the number of chemically feasible molecules ranges from 10^60^ to 10^100^. Despite this vast landscape, computational methods for drug design guide the process towards an intended optimal goal, bypassing the necessity for exhaustive, individual compound evaluation^6^.

In recent years, deep learning has emerged as a promising approach by upscaling the potential of large virtual screening libraries, uncovering new patterns and interconnections to discover new potential bioactive molecules among large databases of molecules through docking, screening, or de novo design. Generative AI can offer rapid access to exhaustive chemical libraries, learning and predicting new binding poses or molecule combinations based on learning patterns, becoming more efficient and accurate over time in developing drug candidates^7^.

Several applications have already been pursued, such as drug repurposing of existing molecules to discover new antibiotics^8^, or drug optimization and design by applying recursive neural networks, autoencoders, generative adversarial networks, and reinforcement learning to generate new molecules while optimizing resources^9–11^.

This study aims to design and implement MedGAN, an optimized and fine-tuned generative architecture using an optimized Generative Adversarial Network (GAN), where two models are trained simultaneously: a generative model (G) that captures the data distribution and a discriminative model (D) that estimates the probability of a sample coming from the training data, with the generative model mapping random noise through multilayer perceptrons and leveraging the backpropagation and dropout algorithms^12^. Molecules are represented as graphs by employing a Graph Convolutional Network (GCN) that handles graph-structured data with various types of relations, such as bonds (edges) between atoms (nodes) and their characteristics, including atom types, chirality, and atom charge (features)^13^. The optimization of the GAN model was performed by employing a Wasserstein GAN (WGAN) with a GCN^14,15^. This approach offers stable training dynamics, effectively overcoming issues such as mode collapse by utilizing the Wasserstein distance as the loss function, a gradient penalty that stabilizes training by ensuring that the critic’s output changes smoothly and not suddenly when the input varies, and the refining of node and edge representations through GCN layers. Studies using WGAN with GCN for molecule generation have already been conducted, achieving small molecular graphs^11,16^. Their size, complexity, and performance are limited by the complexity of the drug-like molecules.

Building on the complex landscape of drug discovery and the emerging role of generative models, a specialized approach can further enhance the efficiency of this process by selecting a single scaffold with known biological interest for drug discovery, therefore addressing a common pattern and reducing the latent space required for learning, achieving a more efficient and accurate generative model^17^.

Quinoline scaffold molecules are ideal candidates because of their distinctive chemical properties and broad biological activities. Their polycyclic aromatic rings and pyridine-like nitrogen are crucial components of a variety of biologically active compounds that allow various electrophilic substitutions, creating a multitude of diverse molecules with distinct stereochemistry that have proven efficacy as therapeutic agents. Recognized for their potential anticancer, anti-inflammatory, antibacterial, and antiviral activities, quinoline and its derivatives offer a rich path for the development of novel drugs (Fig. S1). Numerous mechanisms of action facilitate the inhibition of cell proliferation through cell cycle arrest and apoptosis to disrupt angiogenesis and modulate cell migration, making it an interesting and promising structure for further exploration in drug design^18,19^.

## Results

In our endeavor to develop an effective model for generating novel quinoline scaffolds, we optimized several configurations of the WGAN with the GCN architecture by employing different training parameters in three different models to compare them with a base model and different dataset properties. Input data were structured as graphs with adjacency and feature tensors built from chemical information collected from the training dataset of quinoline molecules. GCN layers analyze the relationships between atoms (nodes) and bonds (edges), learning the intricate patterns that define the molecular structure, while the generator and discriminator compete to produce increasingly realistic molecular structures retaining the quinoline scaffold. The training results led to optimal hyperparameters such as the latent space (256 inputs), optimizer (RMSprop), learning rate (0.0001), and neurons for the Generator and Discriminator (4.092 units) with 63,451,470 and 22,831,617 trainable parameters, respectively. The fine-tuned model with the best performance (Model 3) obtained a 0.25 and 0.62 validity and connectivity scores, respectively, and achieved a 92% success rate in generating quinoline molecules of up to 50 atoms and 7 atom types (C, H, N, O, Cl, S, F), with 93% being novel and 95% unique, preserving properties such as chirality, atom charge, and favorable drug-like properties. Model 3 generated up to 4831 fully connected, novel, and unique quinoline molecules that were absent from the original training dataset. The summarized outcomes are presented in Table 1, providing insights into their respective performances, and samples of the generated molecules are shown in Fig. 2 and Fig. S5.

**Table 1:** MedGAN results. Model performance on molecule generation for the optimization and fine-tuning stages with 95% confidence intervals obtained from 1,000 iterations for each model.

	Base Model	Model 1	Model 2			Model 3		
Latent space	64	64	64			256		
Activation	Tanh/ReLU	Leaky ReLU	Leaky ReLU			Tanh/ReLU		
Optimizer	Adam (1e−5)	RMSprop (1e−5)	RMSprop (1e−4)			RMSprop (1e−4)		
G and D units	512	512	512			4092		
Training iterations*	1000	1000	1000	64	350*	1000	500*	300
Training stage	Optimize (PubChem)	Optimize (PubChem)	Optimize (PubChem)	Fine-tune (ZINC15-II)	Fine-tune (ZINC15-II)	Optimize (PubChem)	Fine-tune (ZINC15-II)	Fine-tune (ZINC15-III)
Validity	0.00 (0.00:0.00)	0.02 (0.02:0.02)	0.14 (0.13:0.14)	0.19 (0.19:0.20)	0.46 (0.46:0.47)	0.16 (0.15:0.16)	0.26 (0.26:0.26)	0.25 (0.24:0.25)
Connected validity	–	0.00 (0.00:0.00)	0.60 (0.59:0.61)	0.47 (0.46:0.47)	0.00 (0.00:0.00)	0.56 (0.56:0.56)	0.65 (0.65:0.65)	0.62 (0.62:0.63)
Quinoline scaffold	–	–	0.96 (0.96:0.97)	0.96 (0.96:0.97)	–	0.98 (0.98:0.98)	0.96 (0.96:0.96)	0.92 (0.91:0.92)
Novelty	–	–	0.65 (0.64:0.66)	0.99 (0.99:1.00)	–	0.61 (0.60:0.62)	0.90 (0.89:0.90)	0.93 (0.92:0.93)
Diversity	–	–	0.84 (0.83:0.84)	0.75 (0.74:0.76))	–	0.91 (0.90:0.91)	0.96 (0.95:0.96)	0.95 (0.94:0.95)
Novel and unique quinolines **	0	0	115	54	0	294	4,831	3,020
PK (Lipinsky)	–	–	–	54 (100.0%)	–	–	4644 (96.1%)	2996 (99.2%)
Toxicity (inactive)	–	–	–	20 (37.0%)	–	–	1009 (22.0%)	935 (31.0%)
SA (Erl)	–	–	–	54 (100.0%)	–	–	4640 (96.0%)	3020 (100,0%)

*Model 2 training stopped at epoch 350 owing to the loss of ability to generate fully connected molecules. Model 3 training stopped at epoch 500 owing to a lack of improvement in both validity and connected validity at this point.

**Each model was evaluated with consecutive runs to generate novel and unique fully connected quinolines not present in the ZINC15 dataset per run until stopped generating additional molecules for 100 consecutive iterations. Pharmacokinetics (PK) were assessed using the Lipinsky rule of 5 (no more than 5 hydrogen bond donors and 10 hydrogen bond acceptors, molecular mass < 500 Da, and logP < 5), and toxicity was assessed as inactive for all 12 targets from the Tox21 pre-trained model; Synthetic Accessibility (SA) score below 6, which indicates that compounds are easily synthesized.

### Optimizer and learning rates

The improved performance of the Root Mean Squared Propagation (RMSProp) over Adaptive Moment Estimation (Adam) algorithms in the task of generating graphs was crucial in our optimization stage. RMSProp employs a moving average of squared gradients to normalize the gradient, which assists in overcoming the issues of aggressiveness and diminishing learning rates. This normalization is particularly beneficial in complex graph generation tasks, where the loss landscape may contain many local minima, making it challenging to find the global minimum. In the case of quinoline generation, this helped RMSProp navigate through these complexities more efficiently. Adam, while also using an adaptive learning approach, may have underperformed because of its momentum component. This component helps accelerate convergence by considering previous gradients, but in the complex task of generating graphs of quinoline molecules, it could lead to overshooting the global minimum or getting stuck in local minima, contributing to RMSProp's superior performance in this specific task^20,21^.

### Activation function

During the optimization stage of quinoline molecule generation, the Leaky Rectified Linear Unit (LeakyReLU) slightly outperformed both the Hyperbolic Tangent (tanh) and Rectified Linear Unit (ReLU) activation functions. The ability of LeakyReLU to allow small negative activations for inputs less than zero potentially mitigates the “dying ReLU” problem, where negative inputs may cause neurons to become inactive. Despite this advantage, the improvement was not significant because the inputs were all binary and positive; therefore, the choice of activation function seemed less critical in this context. During the fine-tuning stage, Model 3, which utilized a combination of tanh and ReLU, achieved the best results among all the model combinations. This outcome suggests that the choice of activation function may have a nuanced impact, depending on the specific stage of training and the characteristics of the task, highlighting the importance of empirical evaluation in selecting the most suitable activation functions for quinoline molecule generation^22,23^.

### Latent dimensions, generator and discriminator units

Increasing the latent dimensions enabled Model 3 to capture more complex variations within the quinoline molecules, enhancing the quality of the generated graphs. Similarly, expanding the number of neurons in the generator and discriminator units allows the model to learn intricate features and improve overall representations. While these enhancements were not pronounced in the optimization stage, they became more significant with a larger dataset in the fine-tuning stage. This indicated a relationship between the volume of data fed into the model and its ability to learn and discern patterns, demonstrating that our baseline model was under parameterized for the task. Nevertheless, it is crucial to recognize the potential risk of overfitting with such complexity, which requires careful balancing using appropriate regularization techniques. Our findings reveal that the increase in these parameters was beneficial up to a certain threshold, reinforcing the importance of finding the optimal balance for the given task^12,24^.

### Data complexity

Because the optimization step laid the groundwork for a minimum dataset structure to work with this model, composed of quinolines with up to 50 atoms and four atom types, the first step of the fine-tuning stage was to determine the capacity of the model to handle increasingly complex training datasets by testing larger molecules, additional atom types, or additional atom properties. Therefore, the original ZINC15 dataset, composed of 4.6 million quinoline molecules with molecular weight between 250 and 500 Daltons and LogP between − 1 and 5, was divided into three subsets of 1 million random quinolines, according to Fig. S2 and Table S1. In subset ZINC15-I, the purpose was to explore an increase in atom length and include halogens, while in subset ZINC15-II, the purpose was to keep the same atom length but include halogens. In subset ZINC15-III, the objective was to enlarge the tensor size to incorporate atom charges, chiral centers, and stereochemistry. Each subset was used to train all the models.

Training collapsed for ZINC15-I, but the remaining subsets allowed training to be performed, which led us to conclude that hyperparameters from model optimization, designed for molecules up to 50 atoms with four atom types, were insufficient for a molecular length of up to 100 atoms. However, they remained effective for molecules with increased complexity, including halogens, charge, chiral centers, and stereochemistry, indicating that model parameters are more sensitive to molecular size than to slight additional complexity in molecule constitution. ZINC-15 subsets II and III were used to proceed with the fine-tuning stage, allowing for an augmentation in the diversity of chemical composition.

### Validity and connected validity

In the optimization stage, where the PubChem dataset of quinoline molecules was used, models 1, 2, and 3 showed a marked improvement over the base model in terms of generating valid chemical structures (Table 1 and Fig. 5). Validity results (percentage of chemically valid molecules among all the generated graphs) for models 2 and 3 achieved scores of 0.14 and 0.16, respectively, leading to their selection for further fine-tuning, where a larger ZINC15 dataset of quinoline molecules was used (subsets ZINC15-II and ZINC15-III, detailed in Methods section). Model 1 was excluded due to its failure to achieve connected validity (a score of 0.00) with ZINC15-II subset, a crucial factor for representing meaningful chemical structures. During the fine-tuning training stage (Table 1 and Fig. 5), Model 2 reached a validity score of 0.19 and a maximum connected validity of 0.47 with the ZINC15-II subset at iteration 64, but lost its ability to generate fully connected molecules during the remaining training, leading to an early termination of its training at iteration 350, despite reaching a validity of 0.46. Conversely, Model 3 demonstrated strong performance with a validity score of 0.26 and maintained a connected validity score of 0.65 until epoch 500 with the ZINC15-II subset, when the training was halted owing to stabilization in performance. For the ZINC15-III subset, Model 2 was unable to generate any molecule (validity 0.00), while Model 3 reached a validity of 0.25 and connected validity of 0.62 at epoch 300, when training was halted due to comparable metric performance with the ZINC15-II subset.

### Novelty and uniqueness

**Figure 1:**
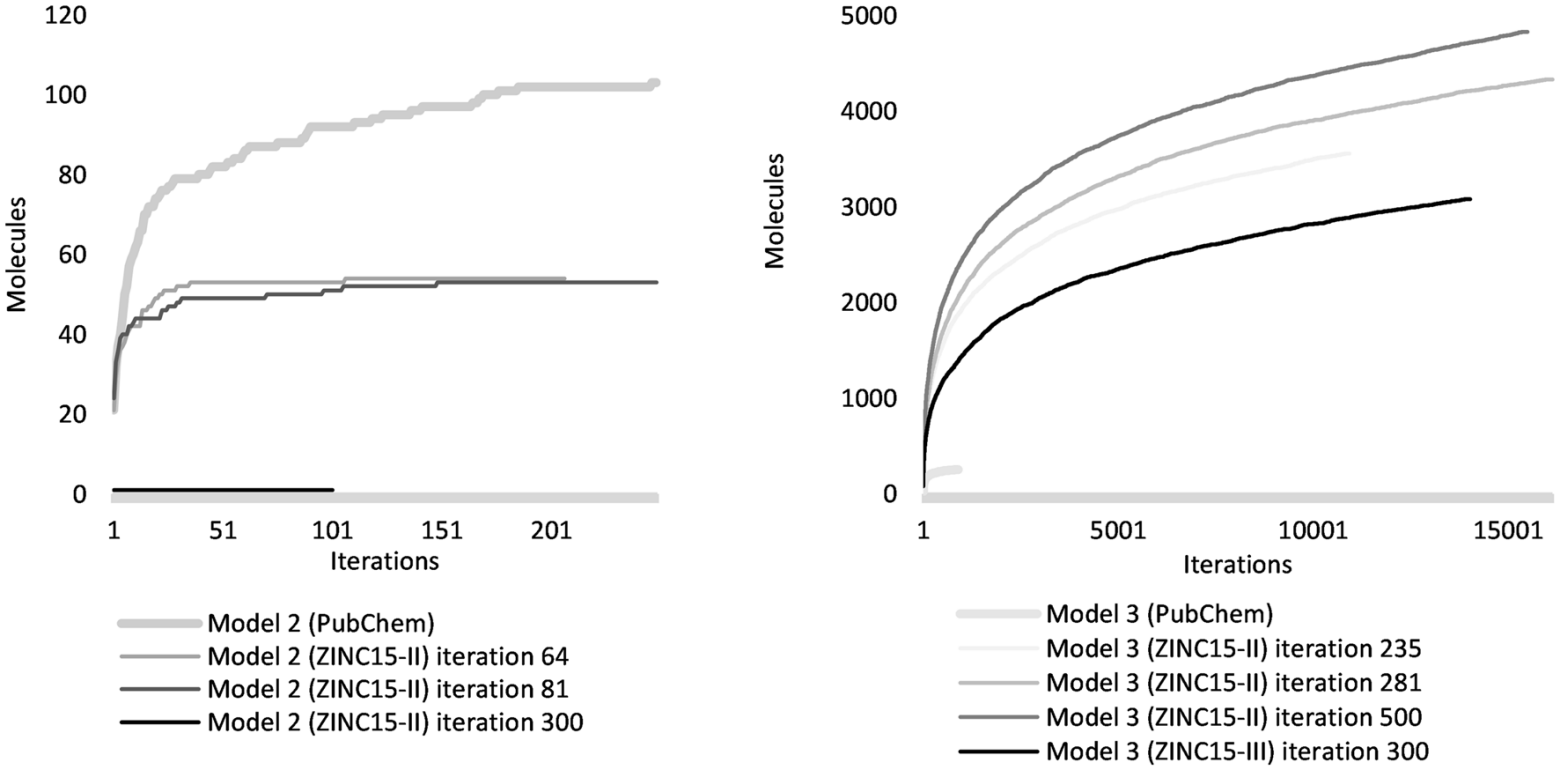
Model generation performance. Molecule generation stopped after 100 consecutive attempts, without producing novel, valid, and unique quinoline molecules. In the optimization stage, Model 3 generated 253 molecules, whereas Model 2 produced 115. During the fine-tuning stage, Model 3 generated 4831 and 3020 unique quinoline molecules when trained with ZINC15-II and ZINC15-III, respectively. In contrast, Model 2 generated 54 molecules before losing its capacity to form fully connected molecules. Model 3, at iterations 235 and 281, had similar validity (0.19), a slightly different result for connected validity (0.64 and 0.68, respectively), and a substantial difference in model performance (22% increase in molecule generation at iteration 281) which indicates a non-linear relation between training performance metrics and molecule diversity.

In the generation of quinoline scaffolds during the optimization stage, models 2 and 3 achieved remarkable quinoline rates of 0.96 and 0.98, respectively. During the fine-tuning stage, Model 3 outperformed Model 2 for the same training subset ZINC15-II by achieving 0.96 in quinoline scaffolds, 0.90 in novel molecules (valid molecules not included in training data among the generated molecules), and 0.96 in unique structures (non-repeated valid molecules generated). For the ZINC15-III subset, Model 3 achieves similar metrics in a shorter time. This novelty metric particularly underscores the capacity of these models to invent new molecular structures, which is an essential aspect in drug discovery and design. To evaluate the capacity of the model to generate new quinoline molecules, 100 graphs were generated in each run until the model was unable to deliver additional graphs that successfully represented fully connected quinoline molecules after 100 consecutive runs. This led to 4831 and 3020 novel and unique quinoline molecules, respectively, achieved by Model 3 with the training subsets ZINC15-II and ZINC15-III (Fig. 1).

### Drug-likeness compliance

Adherence to pharmaceutical guidelines was particularly prominent in Model 3, which generated compounds closely aligned with the Lipinski Rule of 5^25^ in 96.1–99.2% of instances. This model also demonstrated a synthetic accessibility score^26^ below 6 for 96.0–100.0% of the compounds. Furthermore, a significant 22.0–31.0% of the molecules generated by Model 3 showed no toxicity across the 12 predefined targets in a Tox21 pre-trained model (Table S3). The range of inactivity was the lowest for the nuclear androgen receptor-ligand-binding domain (NR-AR-LBD), with values of approximately 50%, and the highest for the nuclear androgen receptor (NR-AR), a crucial transcriptional regulator and therapeutic target in prostate cancer, with values of approximately 100%. The upper and lower limits were achieved for the nuclear androgen receptor (NR-AR), a crucial transcriptional regulator and therapeutic target in prostate cancer (Table S3). In terms of drug discovery, these quinoline molecules might be interesting scaffolds for regulating androgen nuclear receptor activity indirectly through the ligand-binding domain owing to their affinity for interaction, but this should be monitored in terms of toxicity analysis if quinolines are intended for other biological activities. These outcomes adhere to early stage safety assessment practices and highlight the robust and effective approaches applied in our study.

The scaled models learned faster and more effectively. When the ZINC15 larger dataset was used (fine-tune stage), Model 2 started generating quinolines at a very early stage, while Model 3 started generating quinolines at epoch 30 (Fig. 5). Nevertheless, Model 2 lost the capacity to generate fully connected molecules with a larger training dataset. Model 3 revealed a large capacity to produce diverse and unique fully connected quinoline molecules while preserving the atom charge and stereochemistry.

### Molecules generation

**Figure 2:**
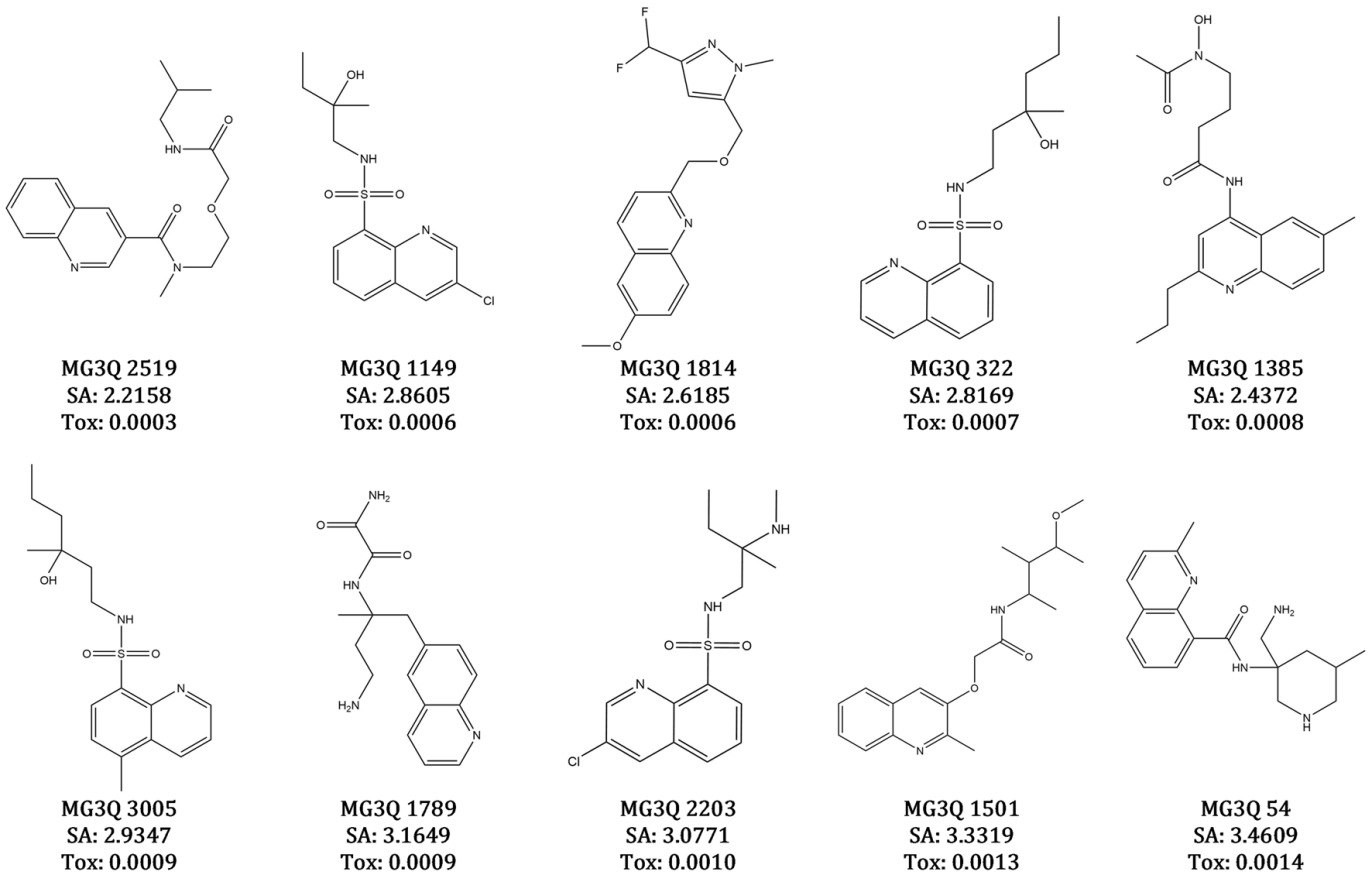
Newly generated quinoline molecules. Ten novel molecules were generated using Model 3, trained with the ZINC15-III subset that passed the Lipinsky rule of five, with SA lower than 6 and not being active for any of the 12 targets on the Tox-21 pre-trained model (top 10 lowest average scores on the classifier model, where Tox values lower than 0.5 means non predicted toxicity for a target).

MedGAN was able to generate thousands of valid, unique, and novel quinoline molecules, most of which have a high probability of being tolerable, non-toxic to humans, and easily synthetized. The following examples show the top 10 quinoline molecules with the lowest average toxicity scores (Tox) for Model 3, trained with the ZINC15-III subset (Fig. 2).

**Figure 3:**
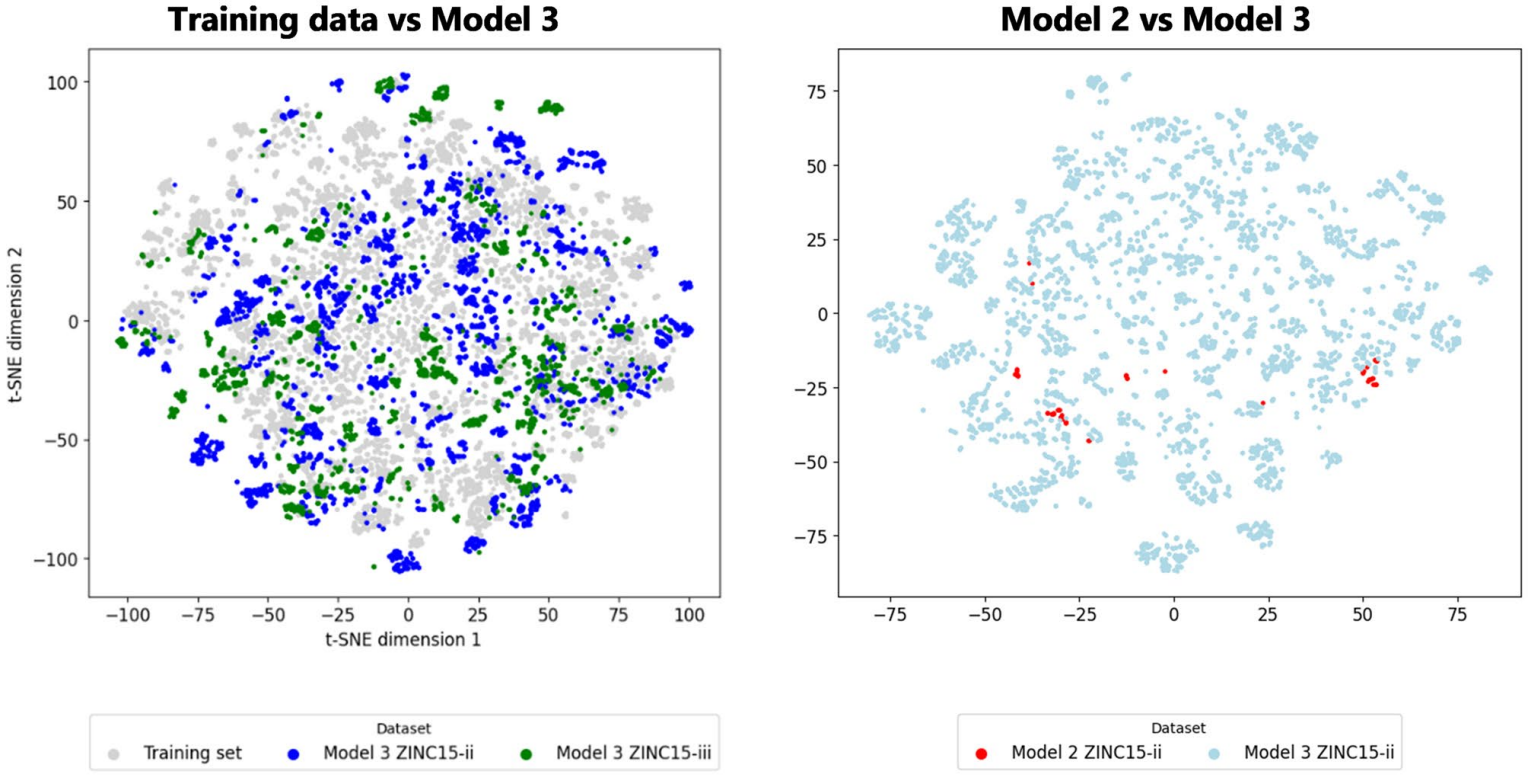
Molecules for t-SNE visualization. 10,000 random samples from the training data (grey) compared with the generated molecules from Model 3 (subsets ZINC15-ii, blue, and ZINC15-iii, green) and Model 2 (subset ZINC15-ii, red).

We also conducted a comparative analysis using the t-distributed Stochastic Neighbor Embedding (t-SNE) technique, which reduces high-dimensional data into two interpretable dimensions, to understand the model’s capacity for generalization (Fig. 3). The analysis indicated distinct clustering patterns, with Model 3 representing a more diverse spread of data points for both subsets when compared to Model 2, and a balanced coverage of the training data chemical space, suggesting its superior ability to explore novel chemical spaces effectively.

## Discussion

Tasks were performed to optimize time and computational resources, ensuring an effective balance between computational complexity and accuracy. This efficiency of task performance sets the stage for our findings.

The sensitivity of the parameters of the generative models to the molecule size and constitution has significant implications. Scaling to larger molecules requires major reoptimization, while scaling to molecules with additional attributes, such as stereochemistry and atom charge, requires less modification, pointing to the complexity of the relationship between molecular structure and the models’ response. MedGAN offers a methodology to search for optimal parameters associated with molecule size and constitution, while using graphs to represent molecules.

The base model and Model 1 were unable to generate quinoline molecules. Models 2 and 3, which were able to generate structurally similar and fully connected molecules, were influenced by factors such as training data size and type, activation function, optimizer, learning rate, latent space, and neuron units of the Generator and Discriminator. Model 2 focused on speed and validity, achieved using the RMSProp optimizer and lower latent space and neuron units in the generator and discriminator. In contrast to Model 3, the superior capacity for generating diverse and fully connected molecules based on the RMSProp optimizer, larger latent space, and higher neuron units highlights the trade-offs inherent in these models. Additionally, testing the generative capacity at different iterations in Model 3 allowed a better understanding of the link between performance metrics and model output, providing new insights into generation effectiveness.

The drug-likeness evaluation for the generated molecules combines efficiency with theoretical feasibility in the selection and development of quinoline-based therapeutics. The comprehensive nature of this analysis reinforces the strategic direction for identifying promising candidates for future novel drugs.

The absence of attention mechanisms and reinforcement learning in this study provides important insights into the optimization and adaptability of generative models for molecular design. Without the focus provided by the attention mechanisms, the model was still able to preserve the quinoline scaffold, indicating a robust learning capacity that did not rely on conditional localized information. The absence of reinforcement learning, typically used for fine-grained optimization through a reward-based paradigm, did not hold back the model's ability to continuously improve validity, novelty, and uniqueness in Model 3. This suggests that a more exploratory and less constrained approach may offer greater flexibility in generating diverse structures, corroborated by previous studies using WGAN and GCN for molecular generation, where a twofold increase in connectivity (reward) also lead to a 6 × decrease in uniqueness^16^. These aspects highlight a potentially more streamlined and adaptable modeling process.

Although there is no direct comparison of MedGAN in the existing literature, an indirect comparison between MolGAN and L-MolGAN without a reinforcement learning strategy (RL) was performed (Table S4). These models with published results were built on a similar architecture of a WGAN with GCN for molecular generation, where the major difference relies on the training data diversity; this study focused on a single scaffold, the optimizer function, where Adam was the choice for both comparators, and different neurons or latent space dimensions due to different molecular lengths. The base model from our work used parameters similar to those of the published models. Compared to the results for L-MolGAN without reinforcement learning, MedGAN improved connectivity (0.620 vs. 0.598) and uniqueness (0.950 vs. 0.197). MedGAN also outperformed it in terms of synthesizability (1.000 vs. 0.950 for MolGAN and 0.290 for L-MolGAN). While indirect comparison has limitations owing to differences in training data, code implementation, and methods used for metrics assessment, it is clear that our model reached the main goal of generating new and diverse molecules^11,16^.

The capacity of MedGAN to generate valid, unique, and large quinoline-scaffold molecules provides a strong foundation for enhancing drug discovery in various therapeutic areas. The specific achievement of optimizing parameters for quinoline-scaffold molecules with up to 50 atoms with seven atom types and preserving the atom’s chirality and charge from graphs provides a concrete path for generating more complex structures in the future and targeting certain biological activities. This specialization not only serves the current scaffold but also provides a framework for other molecular structures, potentially expanding its utility in the broader field of medicinal chemistry.

Using graphs to generate new molecules of therapeutic interest is just a starting point in drug development and has some limitations that can be addressed, as generative models progressively show proven efficacy in generating valid molecules. The interpretability of Generative Adversarial Networks is not straightforward, and training a generative model requires intensive computational power, leading to training constraints that can lead to information loss.

In conclusion, the discoveries made in this study synthesized the complexities of generative modeling with the specific needs of drug discovery. The insights gained extend beyond the immediate findings and offer a nuanced understanding of model behaviors, optimization strategies, and the complexities of molecular design. This study lays the groundwork for future research and development, contributing to a more robust and tailored approach to drug discovery.

## Methods

In this section, we describe the deep learning methodologies and data management strategies employed to generate novel quinoline-like molecules. The central challenge of this study is the enhancement and optimization of Wasserstein Generative Adversarial Network (WGAN) architecture. The goal was to construct a new GAN model capable of generating valid, unique, and complex scaffold-specific molecules without resorting to attention or reinforcement mechanisms. This approach aims to refine the model's performance to its utmost potential, focusing on the learning of specific core patterns, such as the molecular scaffold inherent to the quinoline structure, and laying the processes for further generalization of other scaffold-specific structures or oriented biological activities.

### MedGAN generative model

**Figure 4:**
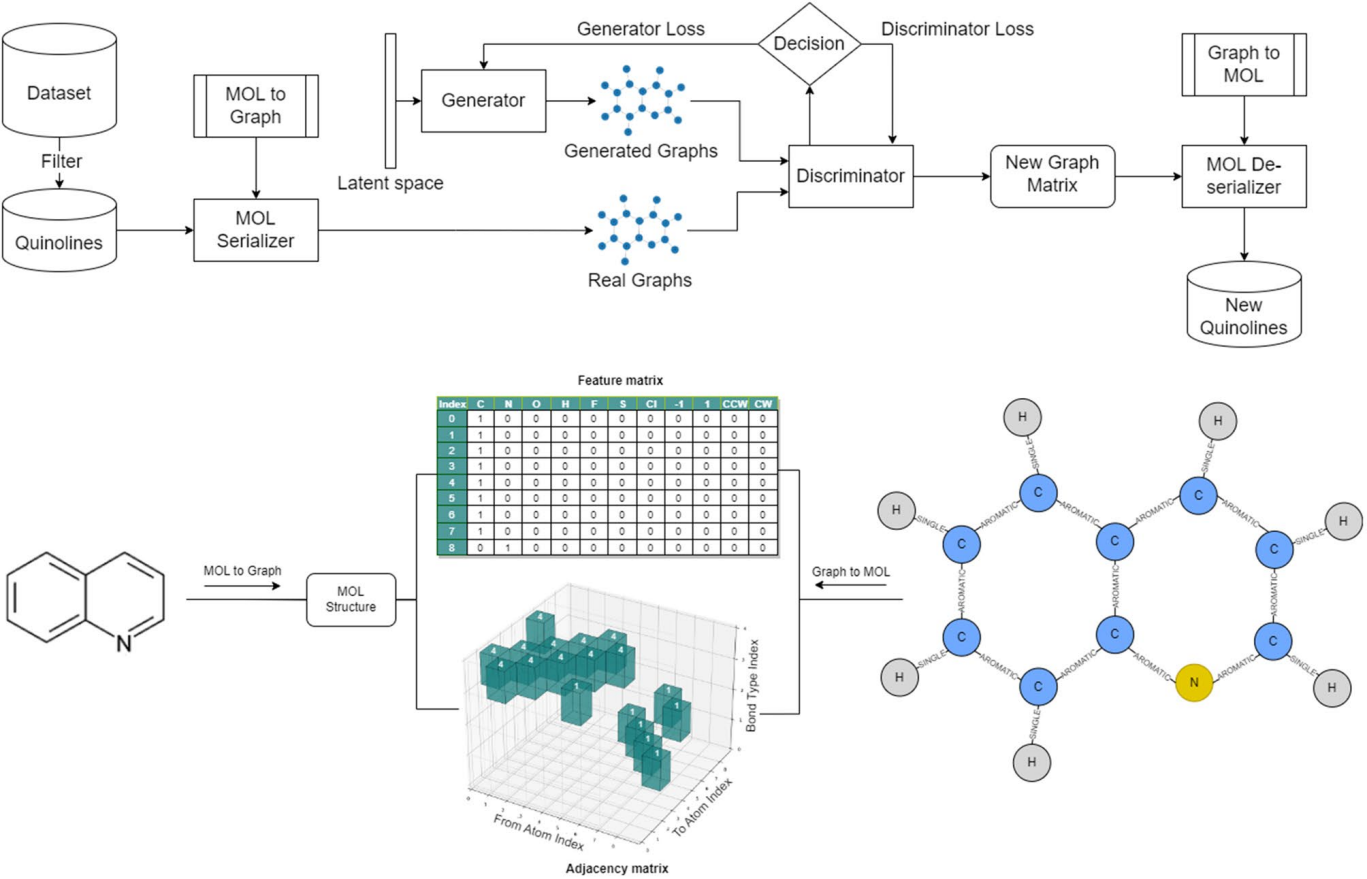
MedGAN Generative Model Architecture. Illustration of the MedGAN model using Wasserstein GANs and Graph Convolutional Networks for molecular graph generation and a sample of a molecule converted to a graph. Beginning with data from PubChem or ZINC15, the process flows through SMILES conversion to Mol, serialization, learning, and deserialization, utilizing specific configurations for optimization, leading to the generation of quinoline-scaffold molecules. Additional details are available in Supplementary Data 3.

GANs can be difficult to train because of issues such as mode collapse, in which the GAN generates a limited diversity of samples, and unstable training dynamics, in which the Generator and Discriminator oscillate without improvement. To overcome these difficulties, Wasserstein GANs have been developed, and together with a Graph Convolutional Network, are the basis of small molecular graph generation (Fig. 4)^12,14,15^. WGAN uses a different type of loss function—the Wasserstein distance (Earth Moving Distance)–accompanied by a gradient penalty function, which provides a smoother gradient for the generator to learn from, reducing the likelihood of training getting stuck, and therefore can lead to more stable and reliable training^14,15^. The WGAN framework is utilized for training both the generator and discriminator in a min–max game, with the Wasserstein distance as the minimized objective. Consequently, the generator learns to progressively create realistic graphs, whereas the discriminator improves its proficiency in distinguishing between genuine and fabricated graphs. The generator network first processes random noise through dense layers to form initial node and edge representations, which are further refined by several Graph Convolutional Network (GCN) layers considering both features and graph connectivity.

After several iterations, these refined representations are processed through another set of dense layers to yield the final node and edge features of the synthetically generated graph with nonhomogeneous relations. The discriminator network employs a GCN to process the input graph. It starts by extracting node and edge features and then feeds them through GCN layers to generate a graph-level representation considering both local features and the overall graph structure. This representation was subsequently passed through a dense layer to produce a scalar value, signifying the authenticity of the input graph.

The base model (Table S2) serves as the foundational architecture for our deep learning approach as a solid starting point for further customization and experimentation, inspired by previous studies in which small molecular graphs were generated^11^. In this study, we did not use reinforcement learning strategies, so that we could focus on the capacity of the model to generate diverse molecular structures, reducing potential overfitting, decreasing the risk of mode collapse, and ensuring simplicity and computational efficiency. Without conditioning for specific chemical properties, the final models may serve a broader set of problems.

### Data pre-processing

Two datasets were collected: PubMed and ZINC15^27,28^. The PubChem dataset was used as a first small subset for model optimization, obtained from a search for “quinoline” keyword and 357,422 valid molecules obtained. The dataset was filtered for true quinoline scaffold molecules and reduced in dimension until it worked with the base model without gradient vanishing, which led to a dimensionality reduction to a maximum of 50 atoms, limited to atom types of carbon, hydrogen, oxygen, and nitrogen (68,881 quinoline molecules). In parallel, the ZINC15 dataset, a collection of 981 million molecules with molecular weight between 250 and 500 Daltons and LogP between -1 and 5, was collected over several days for model fine-tuning after optimization. A filter for the quinoline scaffold in the ZINC15 dataset identified 4,607,029 molecules. The characterization of both datasets is available in Fig. S2 and Table S1.

The atom-type frequency shows that carbon, hydrogen, and nitrogen are present in all molecules, as expected, whereas oxygen is present in almost all molecules. Atoms such as fluorine, sulfur, and chlorine are present in almost 20% of quinoline molecules and can significantly influence the properties of a molecule and its biological activity. Fluorine, owing to its high electronegativity, can alter the polarity, shape, reactivity, and metabolic stability of molecule^29^. Sulfur can participate in different types of bonding, influencing reactivity, and potentially forming metabolites with varying activities^30^. Chlorine enhances lipophilicity and improves drug absorption^31^. Overall, these atoms play key roles in structure–activity relationship studies, significantly affecting the optimization process in medicinal chemistry.

To discern whether the size of molecules or the complexity of their constitution has a greater influence on the optimized parameters of these models and to aid in generating molecules with superior chemical properties that could become lead compounds of interest, the ZINC15 dataset was divided into three distinct subsets according to complexity with 1 million random molecules and C, H, N, O, Cl, S, and F atom types for each: ZINC15-I included quinoline molecules up to 100 atoms; ZINC15-II included molecules up to 50 atoms; ZINC15-III included quinoline molecules up to 50 atoms, atom’s chiral centers, and charge. The subsets are planned to be used sequentially for fine-tuning training in this order.

### Optimization and fine-tuning

A hyperparameter search was conducted on the WGAN base model (Table S2) to ascertain the most effective parameters for optimal model performance. A variety of configurations were tested by varying one parameter at a time while holding the others constant over a span of 100 iterations for each configuration using the PubChem dataset (quinoline scaffold, up to 50 atoms, and C, H, N, and O atom types). The parameters included neuron units, latent space, activation functions, optimizers with various learning rates, regularizers, dropouts, number of generator and discriminator neuron units, and batch sizes. When the upper or lower limits showed no improvement, no further investigation was conducted on this parameter.

The model attempted to generate 100 graphs for each parameter and convert them into molecules. The training progress for the validity metrics and the plausible generated molecules obtained for the parameters that revealed major improvements compared to the base model are available in Fig. S3.

To facilitate a comprehensive comparison, we configured different models alongside our baseline WGAN-GCN model to fine-tune these parameter combinations and determine the optimal configuration of the hyperparameters for high-quality graph output generation (Table 2). The endpoint for the additional models was when a model achieved the generation of valid quinoline molecules, leading to Models 1, 2, and 3.

**Table 2:** Fine-tuned model parameters.

	Base model	Model 1	Model 2	Model 3
Neuron units	128	128	128	128
Latent dimension	64	64	64	256
Activation function	Tanh/ReLU	Leaky ReLU	Leaky ReLU	Tanh/ReLU
Optimizer and learning rate	Adam (1e−5)	RMSprop (1e−5)	RMSprop (1e−4)	RMSprop (1e−4)
Regularizer	Dropout (0.2)	Dropout (0.01)	Dropout (0.5)	Dropout (0.5)
Generator units	512	512	512	4092
Discriminator units	512	512	512	4092
Batch size	32	32	128	32 (512)

**Figure 5:**
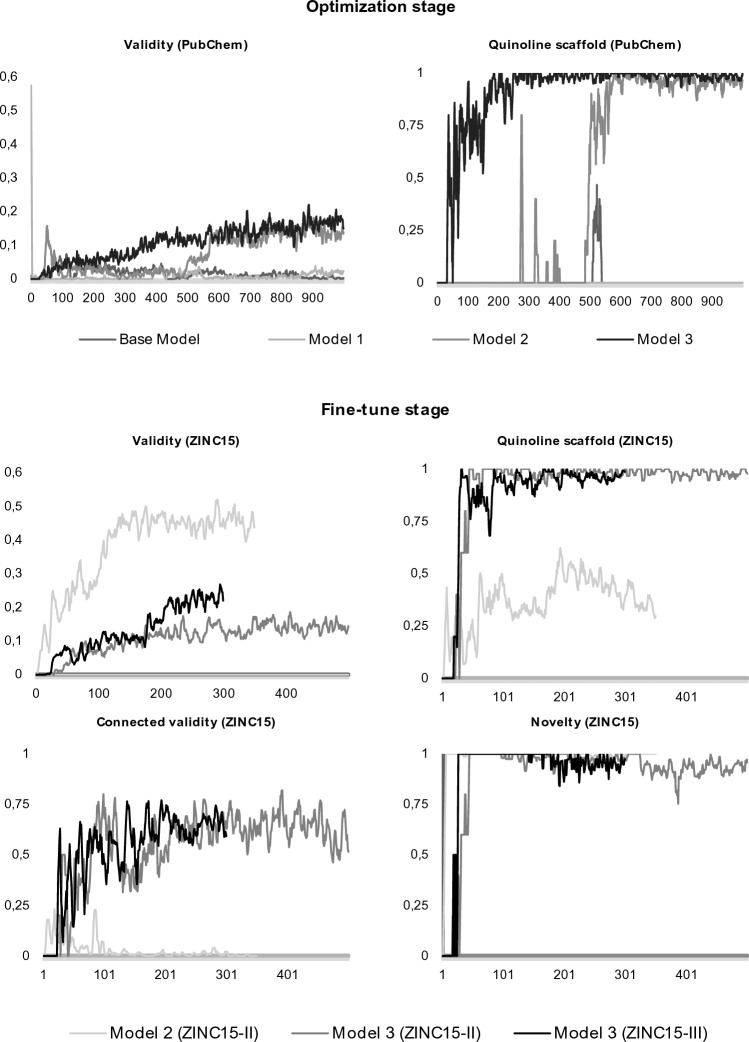
Optimization and fine-tuning performance. The validity and percentage of quinoline molecules were assessed for each model (base, 1, 2, and 3) in the optimization stage (PubChem dataset). Models 2 and 3 revealed the best performance and where further assessed in the fine-tune stage (ZINC15 subsets II and III) for their validity, percentage of quinoline molecules generated, non-fragmented molecules generation (connected validity), and different molecules from the ZINC15 dataset (novelty). For more suitable visualization, a smoothing factor was applied (moving average of five data points).

Model 1 maintained the structure of the base model in several features, which consequently implied an equal number of trainable parameters, namely 6,713,687 for the generator and 578,177 for the discriminator. Variations were observed in the activation function, where Leaky ReLU replaced Tanh + ReLU, and the optimizer was changed to RMSprop with a learning rate of 0.00001. Additionally, the regularizer employs a lower dropout rate of 0.01, which is a shift from the base model rate of 0.2. Model 2, similar to Model 1, retains the structure of the base model, including the trainable parameters. However, it resulted in a more aggressive RMSprop learning rate of 0.0001, accompanied by an increased dropout rate of 0.5. This promotes a more robust defense against overfitting while also offering greater potential for explorative learning. Model 3 has a significantly different form. By increasing the latent dimension to 256, the capacity of the model to represent various features significantly expands. Moreover, the generator and discriminator units increase to 4096, which results in an increase in trainable parameters to 63,451,470 for the generator and 22,831,617 for the discriminator. Despite these changes, the RMSprop optimizer, learning rate of 0.0001, and dropout rate of 0.5 are retained to ensure consistency across models. These models were evaluated for up to 1000 epochs using a hardware configuration consisting of a 24 GB GPU with 32 CPUs and 32 GB of memory. Each model presents a unique blend of hyperparameters, giving us a broad range of configurations to assess their impact on graph output quality (Fig. 5).

### Drug-likeness

Following the capacity of Model 3 to generate quinoline molecules, an assessment was conducted to evaluate their adherence to Lipinski's rule of 5, synthetic accessibility (SA), and predicted toxicity. This multifaceted evaluation allowed us to identify promising candidates that are theoretically viable for further development.

Lipinski's Rule of Five offers a robust method to gauge the bioavailability of the generated molecules, encompassing factors such as molecular weight (less than 500 Da), hydrogen bond donors (not more than 5), hydrogen bond acceptors (not more than 10), and the partition coefficient (logP not greater than 5)^25^.

Synthetic Accessibility (SA) evaluation quantifies the ease with which a molecule can be synthesized in a laboratory setting. It considers the complexity of the molecular structure, the nature of the fragments, how they combine, penalties related to the size and cycles within the molecule, and additional challenges such as synthesizing specific chiral centers. The SA of the generated quinoline molecules was calculated using the Ertl algorithm, providing a nuanced and comprehensive understanding of the synthetic challenges and potential barriers to laboratory production^26^.

To ensure the safety profile of the generated molecules, toxicity was predicted using DeepChem's pretrained model on Toxicology in the 21st Century (Tox21) dataset, which comprises 12,060 training samples and 647 test samples with 801 features that represent chemical descriptors (molecular weight, solubility, or surface area) and 272,776 features that represent chemical substructures for machine learning applications^32^. For each sample, 12 binary labels represented the outcome (active/inactive) of 12 different toxicological experiments. This tool facilitates the assessment of potential toxicity risks based on molecular structure, offering key insights into the suitability of each molecule for pharmaceutical development. The program systematically evaluated the activity of compounds across 12 specific biological targets. Among these targets are five nuclear receptors: Androgen Receptor (AR), which is involved in male sexual development; Estrogen Receptor (ER), which is key to female reproductive health; Peroxisome Proliferator-Activated Receptor Gamma (PPARγ), a regulator of metabolism; Aryl Hydrocarbon Receptor (AhR), a sensor of environmental toxins; and Thyroid Receptor Beta (TRβ), which is essential for thyroid hormone balance. Additionally, five stress response pathways were identified: ATPase family, AAA domain-containing 5 (ATAD5), an indicator of DNA damage; p53, a tumor suppressor; Heat Shock Element (HSE), a sensor for protein damage; Antioxidant Response Element (ARE), key in oxidative stress response; and Estrogen Response Element (ERE), involved in estrogen signaling. Two other essential targets, the Mitochondrial Membrane Potential, indicating mitochondrial health, and the estrogen receptor (ER), already covered by nuclear receptors, provide further insights into cell health and endocrine function, respectively. Through analysis of the generated quinoline molecules using DeepChem's Tox21 model, this comprehensive evaluation forecasts potential toxic effects across these carefully selected targets, allowing for the elimination of compounds that could present significant risks in later stages of drug development^32^.

The Tox21 model was trained using a convolutional graph method with data constraints tailored to molecules with a maximum of 50 atoms, including C, H, N, O, Cl, S, or F atom types (5.509 training samples, 714 validation samples, and 704 test samples). This constraint is harmonized with the generative model specifications. Over the course of 100 epochs, the training process exhibited a consistent decline in loss and enhancement in the area under the curve (AUC) for both the training and validation sets. The model stabilized at a final loss of 0.135, a training AUC of 0.987, and a validation AUC of 0.741 (Fig. S4). This trend underscores the ability of the model to learn the underlying pattern in the data, culminating in a reliable predictive model, with performance being monitored and optimized for both training and validation.

### Supplementary Information

Supplementary Information.

## Data Availability

The datasets and code used for training in this study are available from the MedGAN GitHub repository (https://github.com/bmacedo111/MedGAN/).
