# Supplementary material for: MedGAN: optimized generative adversarial network with graph convolutional networks for novel molecule design
Source: Sci Rep. 2024 Jan 12;14:1212. doi: 10.1038/s41598-023-50834-6 (PMC10786821; doi:10.1038/s41598-023-50834-6)
Supplement: Supplementary file 1 — Supplementary Information. [file 41598_2023_50834_MOESM1_ESM.docx]

**Supplementary data**

**S1. Examples of quinoline-based medicines**

**Fig. S1 | Structural representations of quinoline scaffold and derivatives.** Quinoline is a foundational scaffold in medicinal chemistry, underlying its potential in anticancer, anti-inflammatory, antibacterial, and antiviral activities.

**S2. Dataset characterization**

**Fig. S2 | PubChem and ZINC15 datasets.** Characterization for quinoline-scaffold molecules

**Table S1 | Dataset characterization.** ZINC15 subsets from the original ZINC15 dataset composed of 4.6 million quinolines.

ZINC15 data was obtained from the website <https://zinc15.docking.org/tranches/home/> with a filter for Drug-Like properties (981 million substances). The filters for quinoline molecules and their conversion to SMILES are available in a CSV file, and the conversion to graphs is available in NumPy compressed files for adjacency and node features, all in the GitHub repository (<https://github.com/bmacedo111/MedGAN/tree/main/data>).

**S3. Model architecture**

**Input**

**Latent Vector (*z)***

A random vector of shapes according to the latent dimension was sampled from a Gaussian distribution.

$$z\sim N\left( 0,I \right)$$

**Architecture**

**Dense Layers**

The latent vector (*z)* is passed through multiple dense layers, and each layer applies a hyperbolic tangent *(tanh)* function:.

$$y=\frac{e^{Wx+b}-e^{-\left( Wx+b \right)}}{e^{Wx+b}+e^{-\left( Wx+b \right)}}$$

$$\text{where} W\mathrm{and}b are the weight and bias of the layer, respectively$$

**Adjacency Matrix Generation (A)**:

The final dense layer was reshaped and symmetrized into an adjacency tensor. *Softmax* is applied to the tensor along the axis=1.

$$A_{ij}=\frac{e^{x_{adjacency,ij}}}{\sum_{j=1}^{N} e^{x_{j}}}$$

**Feature Matrix Generation (F)**:

The final dense layer is reshaped into a feature tensor. *Softmax* is applied to the tensor along the axis=2.

$$F_{ij}=\frac{e^{x_{features,ij}}}{\sum_{j=1}^{N} e^{x_{j}}}$$

**Discriminator**

**Graph Convolution Layers**

These layers performed Relational Graph Convolutions (ReLU in Model 3 and LeakyReLU in Model 2).

$$Xout=ReLU\left( Aggregate\left( A,F \right)W \right)=max(0,Aggregate\left( A,F \right)W)$$

$$Xout=LeakyReLU\left( Aggregate\left( A,F \right)W \right), where LeakyReLU\left( x \right)= \left\{ \begin{aligned} x if x> 0 \\ \alpha x if x \leq0 \end{aligned} \right.$$

where Aggregate(*A*,*F*) is a function that takes the adjacency matrix *A* and the feature matrix *F* as inputs and aggregates information from neighboring nodes. This aggregated information is then passed through a weighted linear transform, *W*, and activated by the ReLU function. Constant α determines the "leakiness" of the function in LeakyReLU.

**Dense Layers**:

The output of the graph convolutional layers is passed through dense layers with activation as the ReLU.

$$y=ReLU\left( Wx+b \right)=max(0,Wx+b)$$

A dropout layer was applied with a dropout rate of.

**Final Dense Layer**:

Outputting a single scalar value representing the "realness" of the input graph.

**Loss Functions**

**Discriminator Loss**

$$D_{Loss}=D_{Generated}-D_{Real}+ \lambda\times GP$$

**Generator Loss**

$$G_{Loss}={- D}_{Generated}$$

**Gradient Penalty**

$$GP=\left( 1-|\left| \nabla A \right|| \right)^{2}+ \left( 1-|\left| \nabla F \right|| \right)^{2}$$

||∇Α|| and ||∇F|| are the norms of the gradients of the discriminator’s output with respect to Adjacency and Feature matrix, respectively.

**Optimizer: RMSProp**

The RMSProp algorithm is used to update the weights *W* and biases *b* for both the discriminator (D) and generator (G), aiming to minimize their respective loss functions with a gradient as follows:

$$g_{D}=\nabla_{W,b}DLoss$$

$$g_{G}=\nabla_{W,b}GLoss$$

Update the moving average of the past squared gradients for both the Discriminator and the Generator.

$$v_{D,G}=\beta v_{D,G}+\left( 1-\beta\right)g_{D,G}2$$

Update the weights W and biases b for both the Discriminator and the Generator:

$$W_{D,G}=W_{D,G}-\alpha\frac{g_{D,G}}{\sqrt{v+\epsilon}}$$

$$b_{D,G}=b_{D,G}-\alpha\frac{g_{D,G}}{\sqrt{v+\epsilon}}$$

where,

*α* is the learning rate.

*β* is the decay rate.

*ϵ* is a small constant to prevent division by zero.

**S4. Hyperparameter search**

**Table S2 | Hyperparameter search.** The configurations that offered the most promising results compared with the baseline model were identified based on their validity performance.

**Fig. S3 |** **Validity performance using various optimized parameters.** The percentage of valid generated molecules was assessed, and better results were observed for the LeakyReLU activation function, RMSProp optimizer with a lower learning rate, and increased latent dimensions and neuron units for the generator and discriminator.

**S5. Tox21 Model training and results**


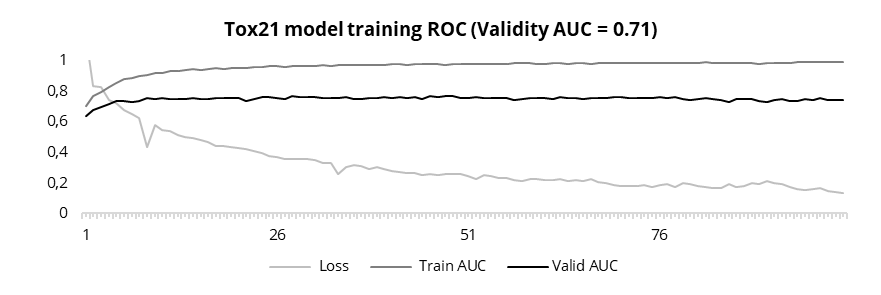


**Fig. S4 | Training Progression of the Tox21 Model.** Convolutional Graph constraints were applied to ensure the same molecular size and atom type.

**Table S3 | Toxicity.** Models 2 and 3 generated molecules were evaluated with the pre-trained tox21 model for 12 targets and the results show the percentage of non-toxic compounds with mean and median values.

* Random sample of 10,000 molecules

**S6. Molecules generated**

**Fig. S5 |** Samples of molecules generated with Model 3 trained with subset ZINC15 III

**S7. Model comparison**

**Table S4 | Comparative results.** Results for MedGAN, MolGAN, and L-MolGAN.

* For both models the comparative results do not include a reinforcement learning strategy (𝜆 = 1.0 in MolGAN and 𝜆 = 0.0 in L-MolGAN).
